# Are viral loads in the febrile phase a predictive factor of dengue disease severity?

**DOI:** 10.1186/s12879-024-10152-2

**Published:** 2024-11-05

**Authors:** Shashika Dayarathna, Heshan Kuruppu, Tehani Silva, Laksiri Gomes, N. L. Ajantha Shyamali, Chandima Jeewandara, Dinuka Ariyaratne, Shyrar Tanussiya Ramu, Ananda Wijewickrama, Graham S. Ogg, Gathsaurie Neelika Malavige

**Affiliations:** 1 Allergy Immunology and Cell Biology Unit, Department of Immunology and Molecular Medicine, University of Sri Jayewardenepura, Nugegoda, Sri Lanka; 2 Faculty of Medicine, General Sir John Kotelawala Defence University, Rathmalana, Sri Lanka; 3 National Institute of Infectious Diseases, Angoda, Sri Lanka; 4 MRC Human Immunology Unit, MRC Weatherall Institute of Molecular Medicine, University of Oxford, Oxford, UK

## Abstract

**Background:**

As many studies have shown conflicting results regarding the extent of viraemia and clinical disease severity, we sought to investigate if viraemia during early dengue illness is associated with subsequent clinical disease severity.

**Methods:**

Realtime PCR was carried out to identify the dengue virus (DENV serotype), in 362 patients, presenting within the first 4 days of illness, from 2017 to 2022, in Colombo Sri Lanka. To characterize subsequent clinical disease severity, all patients were followed throughout their illness daily and disease severity classified according to WHO 1997 and 2009 disease classification.

**Results:**

263 patients had DF, 99 progressed to develop DHF, and 15/99 with DHF developed shock (DSS). Although the viral loads were higher in the febrile phase in patients who progressed to develop DHF than in patients with DF this was not significant (*p* = 0.5). Significant differences were observed in viral loads in patients infected with different DENV serotypes (*p* = 0.0009), with lowest viral loads detected in DENV2 and the highest viral loads in DENV3. Sub-analysis for association of viraemia with disease severity for each DENV serotype was again not significant. Although those infected with DENV2 had lower viral loads, infection with DENV2 was significantly associated with a higher risk of developing DHF (*p* = 0.011, Odds ratio 1.9; 95% CI 1.164 to 3.078). Based on the WHO 2009 disease classification, 233 had dengue with warning signs (DWW), 114 dengue without warning signs (DWoWS), and 15 had severe dengue (SD). No significant difference was observed in the viral loads between those with SD, DWW and DWoWS (*p* = 0.27).

**Conclusions:**

Viral loads were significantly different in the febrile phase between different DENV serotypes, and do not appear to significantly associate with subsequent clinical disease severity in a large Sri Lankan cohort.

**Supplementary Information:**

The online version contains supplementary material available at 10.1186/s12879-024-10152-2.

## Background

Dengue, which was named as one of the top ten threats to global health by the WHO, is the most rapidly increasing mosquito borne viral infection [1]. As it is a climate sensitive infection, due to the rise in global temperatures, it is predicted that burden of dengue is likely to further increase, resulting in potential overwhelming of the health-care systems in further areas and countries [2]. As there is no effective treatment for dengue, all patients with a suspected DENV infection are closely monitored for early detection of complications for timely interventions in the form of meticulous fluid management [3].

While most DENV infections result in asymptomatic or mild illness, some individuals develop vascular leakage resulting in pleural effusions, ascites, shock and other complications such as bleeding and organ dysfunction [3]. The reasons for occurrence of vascular leakage and other complications in some individuals are unknown, but secondary dengue, pregnancy, presence of comorbidities such as diabetes, obesity and chronic kidney disease are known risk factors [4–6]. A dysfunctional innate immune response that results in an impaired antiviral immunity, with an enhanced proinflammatory response, presence of poor quality non-neutralizing antibodies and a sub-optimum T cell response are known to contribute to severe disease [7, 8].

Although severe clinical disease manifestations occur due to an aberrant and suboptimum immune response, the dengue virus (DENV) is known to lead to induction of many inflammatory mediators and therefore, directly contributes to disease pathogenesis [9]. Therefore, targeting the DENV and inhibiting viral replication has been one of the main strategies for developing a treatment for dengue [10]. However, many studies have shown conflicting results regarding the extent of viraemia and clinical disease severity. In a recent very large study from Vietnam, it was shown that higher plasma viraemia increased the risk of severe disease, hospitalization and plasma leakage, irrespective of the infecting serotype and the immune status [11]. However, they also show that although the viral loads were highest for DENV1 and lowest with infections with DENV2, infection with DENV2 was associated with a higher risk of developing severe dengue [11]. Another study from Vietnam showed that viral loads during early illness was significantly less in those with secondary dengue, although secondary dengue is an important risk factor for severe disease [12]. A study from Indonesia showed that DENV3 was associated with a higher risk of plasma leakage compared to other serotypes and that there was a trend towards higher viraemia in those who develop severe dengue [13]. Studies from Colombia and India showed that the viral loads had no relationship with clinical disease severity [14, 15].

The contradictory findings in different studies could be due to different viral dynamics based on timing, different genotypes of the virus, differences in host responses based on genetic predisposition and ethnicities. As many antiviral trials for dengue failed to show efficacy so far, and as many trials are underway, it would be important to understand if viral loads during early illness associate with subsequent disease severity in different countries and in different populations. In this study, we have characterized the viral loads during early illness in patients presenting to a large tertiary care hospital in Sri Lanka, over a period of 5 years, to understand the relationship between viral loads, different DENV serotypes and clinical disease severity.

## Methods

### Patients with acute dengue infection

Adult patients (*n* = 412) with suspected acute dengue were recruited from the National Institute of Infectious Diseases, Sri Lanka from September 2017 to September 2022 following informed written consent to the study. Blood samples were collected from each patient, during the first four days since onset of symptoms (days of illness ≤ 4 days), which was during the febrile phase. A second blood sample could only be collected from 149 patients between day 5 to 7 of illness for dengue antibody assays, as some patients did not consent to obtaining a second blood sample as they were bled several times each day for routine investigations. Any patient who had features of plasma leakage at the time of recruitment, were excluded from the study, as we wished to assess the relationship of viral loads with subsequent disease severity, including development of plasma leakage. To characterize clinical disease severity, all patients were followed throughout their illness daily, and all clinical and laboratory features were recorded. 44 patients did not require admission, while all other patients were admitted to hospital. Ultrasound scans were done in all patients at the time of admission to detect the presence of fluid leakage and repeated daily until the patient recovered, to detect the presence of any fluid leakage. If the patients showed a rise in the haematocrit, with subsequent reduction in platelet counts (these parameters were monitored on a daily basis in all patients), ultrasound scans were performed to detect pleural effusions and ascites.

### Determining the DENV serotype and viral loads

Blood samples were centrifuged, and the obtained serum samples were aliquoted and stored at -80 °C to avoid repeated freeze-thawing. Viral RNA was extracted from the serum using QIAmp Viral RNA Mini Kit (Qiagen, USA, Cat: 52906). cDNA transcription was performed with a high-capacity cDNA reverse transcription kit (Applied Biosystems, USA, cat: 4368814). Oligonucleotide primers, dual labelled probes for DEN 1 to 4 serotypes (Life Technologies, India) and TaqMan Multiplex Master Mix (Applied Biosystems, USA, Cat: 4461881) were used for the multiplex quantitative real-time PCR in ABI 7500 real time PCR system (Applied Biosystems, USA) as previously described [16]. The standard curve was generated using four gBlock fragments with known copy numbers (Integrated DNA Technologies, USA).

### Dengue IgM and IgG antibody assays

Dengue antibody assays were performed in 149 patients, in whom a second sample could be collected between day 5 to 7 of illness, using a commercial capture-IgM and IgG Enzyme- Linked Immunosorbent Assay (ELISA) (Panbio, Australia). Results were interpreted according to the manufacturer’s instructions. According to the WHO 2011 criteria, patients with an IgM: IgG ratio of > 1.2 were classified as having primary dengue, while patients with IgM: IgG ratio of < 1.2 were categorized under secondary dengue [3].

### Statistical analysis

Statistical analysis was done using GraphPad Prism version 9.5.1 (Dotmatics, California, USA). As the data were not normally distributed, differences in the viral loads for different clinical disease severity and serotypes were compared using the Mann-Whitney U test (two tailed). The Kruskal-Wallis test was used to compare the viral loads between different serotypes, when more than one parameter was compared. Spearman rank order correlation coefficient was used to evaluate the correlation between variables including the association between viral loads and laboratory parameters.

## Results

### Patient characteristics

The 412 adult patients were recruited on a median duration of day 3 (IQR 3 to 4 days) since onset of illness. The DENV serotype was identified in 362 (87.9%) patients who were included in the analysis. 50 patients tested negative in qPCR serotyping and therefore were excluded from the analysis. In order to determine the relationship between viral loads in the febrile phase and subsequent clinical disease severity, patients were classified as having DF or DHF, according to the WHO 1997 criteria [3]. Analysis was also carried out by classifying them as having dengue with warning signs (DWW), without warning signs (DWoWS) and with severe disease (SD) according to the WHO 2009 disease classification guidelines [17]. The clinical and laboratory characteristics of patients when classified as DF/DHF, and DWW, DWoWS and SD are shown in Tables 1 and 2. The mean age of those who were recruited to the study was 33.02 years (SD ± 13.47) and median age 29 years. 234 (56.8%) patients were in the 20- to 40-year-old age group. There was no correlation with the age and the viral loads during the febrile phase of illness (Spearman’s *R*=-0.07, *p* = 0.21).

**Table 1:** Clinical and laboratory features of patients who were classified as having DHF and DF based on the WHO 1997 disease classification

Clinical findings	DF (*n* = 263) *N*%		DHF (*n* = 99) *N*%	
Fever	243	(92.4)	96	(97.0)
Arthralgia	142	(54.0)	71	(71.7)
Myalgia	137	(52.1)	64	(64.6)
Flushing	13	(4.9)	7	(7.1)
Rashes	2	(0.8)	2	(2.0)
Itching	2	(0.8)	1	(1.0)
Abdominal pain	51	(19.4)	38	(38.4)
Vomiting	61	(23.2)	31	(31.3)
Diarrhoea	75	(28.5)	40	(40.4)
Pleural effusion	0		14	(14.1)
Ascites in hepatorenal pouch	0		81	(81.8)
Hepatomegaly	1	(0.4)	1	(1.0)
Petechiae	2	(0.8)	1	(1.0)
Ecchymoses	0		0	
Epistaxis	0		2	(2.0)
Haematemesis	0		0	
Malena	1	(0.4)	0	
Bleeding from the site of venepuncture	0		0	
Vaginal bleeding	4	(1.5)	7	(7.1)
**Lowest platelet count /mm** ^**3**^				
< 20,000	8	(3.0)	51	(51.5)
20,000 to 50,000	50	(19.0)	35	(35.4)
50,000 to 100,000	92	(35.0)	8	(8.1)
> 100,000	110	(41.8)	4	(4.0)
**Lowest WBC count cells/µl**				
< 2000	38	(14.4)	15	(15.2)
2000 to 5000	195	(74.1)	76	(76.8)
> 5000	28	(10.6)	7	(7.1)

**Table 2:** Clinical and laboratory features of patients who were classified as having severe dengue (SD), dengue with warning signs (DWW) and dengue without warning signs (DWoWS) according to the WHO 2009 disease classification

Clinical findings	SD (*n* = 15) *N*%		DWW (*n* = 233) *N*%		DWoWS (*n* = 114) *N*%	
Fever	14	(93.3)	223	(95.7)	102	(89.5)
Arthralgia	10	(66.7)	152	(65.2)	51	(44.7)
Myalgia	9	(60.0)	143	(61.4)	41	(36.0)
Flushing	1	(6.7)	15	(6.4)	4	(3.5)
Rashes	1	(6.7)	3	(1.3)	0	
Itching	0		3	(1.3)	0	
Abdominal pain	3	(20.0)	84	(36.1)	2	(1.8)
Vomiting	4	(26.7)	88	(37.8)	0	
Diarrhoea	4	(26.7)	93	(39.9)	18	(15.8)
Pleural effusion	0		14	(6.0)	0	
Ascites in hepatorenal pouch	8	(53.3)	73	(31.3)	0	
Hepatomegaly	0		2	(0.9)	0	
Petechiae	0		2	(0.9)	2	(1.8)
Ecchymoses	0		0		0	
Epistaxis	0		2	(0.9)	0	
Haematemesis	0		0		0	
Malena	0		1	(0.4)	0	
Bleeding from the site of venepuncture	0		0		0	
Vaginal bleeding	2	(13.3)	7	(3.0)	2	(1.8)
**Lowest platelet count /mm** ^**3**^						
< 20,000	7	(46.7)	52	(22.3)	0	
20,000 to 50,000	3	(20.0)	82	(35.2)	0	
50,000 to 100,000	1	(6.7)	69	(29.6)	30	(26.3)
> 100,000	3	(20.0)	30	(12.9)	81	(71.1)
**Lowest WBC count cells/µl**						
< 2000	3	(20.0)	36	(15.5)	14	(12.3)
2000 to 5000	8	(53.3)	182	(78.1)	81	(71.1)
> 5000	3	(20.0)	15	(6.4)	17	(14.9)

According to the WHO 1997 guidelines, those who had features of plasma leakage (pleural effusions or ascites along with platelet counts < 100,000 cells/mm^3^ or a rise in haematocrit of > 20% were classified as having DHF. None of the patients had pericardial effusions. As daily ultrasound scans were done in all patients, to determine the presence of fluid leakage and when they developed fluid leakage, as per the National Management guidelines, serum albumin levels were not done [18]. Patients with DHF, who had a narrowing pulse pressure ≤ 20 were classified as having shock. Accordingly of the 362 PCR positive patients, 263 patients had DF, 99 progressed to develop DHF and of them 15 developed shock (DSS). Among the 362 qPCR positive patients, 231 (63.8%) were of males and 131 (36.2%) were of females, and 63 (27.3%) of males developed DHF vs. 36 (27.5%) females. Overall, the viral loads were significantly higher (*p* = 0.0021) in males (median 5.72 × 10^4^ copies/µl, IQR: 1.14 × 10^3^ copies/µl to 2.15 × 10^6^ copies/µl.), compared to females (median 5.47 × 10^3^ copies/µl, IQR: 9.39 × 10^1^ copies/µl to 2.90 × 10^5^ copies/µl). However, although males with DF had significantly higher viral loads than females with DF (*p* = 0.003), no such difference was seen in males and females with DHF (*p* = 0.34). Based on the WHO 2009 disease classification, 233 had DWW, 114 DWoWS, and 15 had SD.

### Viral loads at febrile phase and relationship with clinical disease severity based on WHO 1997 disease classification

**Fig. 1:**
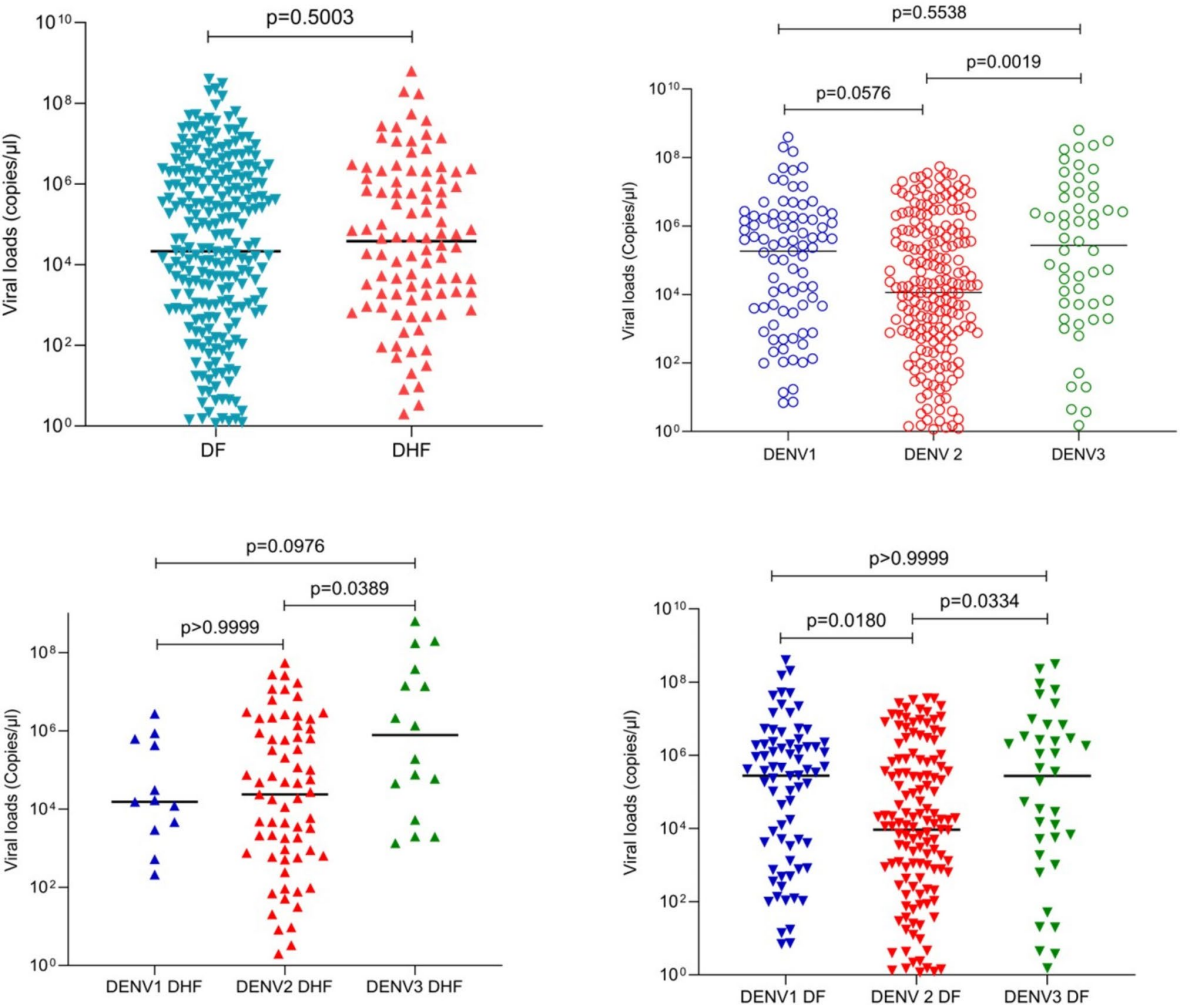
Viral loads in patients with varying clinical disease severity and infected with different DENV serotypes. Viral loads in serum samples were measured by qPCR in those with DF (*n* = 261) and in those who progressed to DHF (*n* = 98), during the febrile phase (**A**). Viral loads were compared in patients infected with different DENV virus serotypes, DENV1 (*n* = 91), DENV2 (*n* = 214) and DENV3 (*n* = 54) (**B**) in those with DHF (**C**) and DF (**D**) infected with different virus serotypes. The Mann-Whitney test was used to compare the differences in viral loads between those with DF and DHF, while Kruskal-Wallis test was performed to compare the differences in viral loads in those infected with different serotypes. All tests were two sided. Data are presented as median values +/- interquartile ranges as appropriate

214 (59.1%) of patients were infected with DENV2, 91 (25.1%) with DENV1 and 54 (14.9%) with DENV3, while DENV4 was not detected in any of the patients. Three patients were found to be infected with two DENV serotypes: DENV1 and DENV2 (*n* = 2) and DENV2 and DENV3 (*n* = 1) and were not included in the analysis. Although the viral loads were higher in the febrile phase in patients who progressed to develop DHF (median 3.80 × 10^4^ copies/µl, IQR: 1.36 × 10^6^ copies/µl to 1.24 × 10^3^ copies/µl) than in patients with DF (median 2.13 × 10^4^ copies/µl, IQR: 1.58 × 10^6^ copies/µl to 2.64 × 10^2^ copies/µl), this was not significant (*p* = 0.50) (Fig. 1A). There were no significant differences in viral loads during the febrile phase in those who progressed to develop DSS vs. DHF and DF (*p* = 0.79).

However, significant differences were observed in viral loads in patients infected with different DENV serotypes (*p* = 0.0009) (Fig. 1B). The lowest viral loads were detected in those infected with DENV2, while the highest viral loads were observed in those infected with DENV3. Although those infected with DENV2 had lower viral loads, infection with DENV2 was significantly associated with a higher risk of developing DHF (*p* = 0.0106, Odds ratio 1.9; 95% CI 1.164 to 3.078), when compared to other serotypes.

Significant differences were seen in the febrile phase in viral loads of patients infected with different viral serotypes, who subsequently progressed to develop DHF (*p* = 0.0331), (Fig. 1C) or DF (*p* = 0.004), (Fig. 1D). Although not significant, those who were infected with either DENV2 or DENV3 and subsequently progressed to DHF, had higher viral loads in the febrile phase than those who had DF. In contrast, in those infected with DENV1, those who progressed to DHF, had lower viral loads in the febrile phase than those who had DF. Due to the differences in the viral loads seen between different serotypes, we carried out a sub-analysis to see if viral loads were associated with disease severity for each DENV serotype. There were no differences in the viral loads in those with DF and DHF in those infected with DENV1 (*p* = 0.22), DENV2 (*p* = 0.21) or DENV3 (*p* = 0.25).

**Fig. 2:**
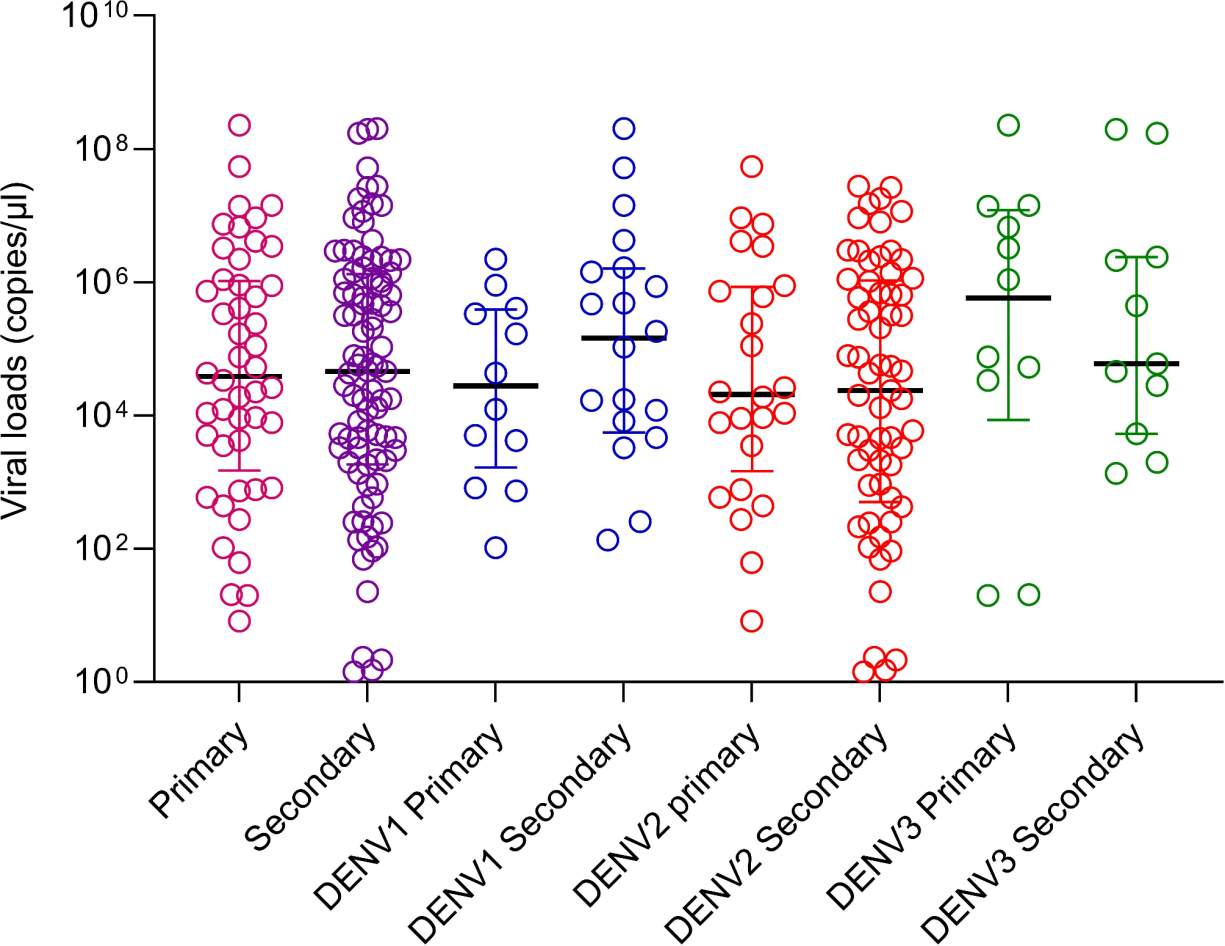
Viral loads in patients with primary and secondary dengue. Viral loads in serum samples were measured by qPCR in those primary (*n* = 48) and secondary (*n* = 96) dengue. They were also compared in those with primary and secondary dengue, infected with different DENV serotypes. The Mann-Whitney test was used to compare the differences in viral loads between those with primary and secondary dengue. All tests were two sided. Data are presented as median values +/- interquartile ranges as appropriate

Although a second blood sample was obtained in 149 of the initial cohort of patients, as the virus serotype could only be determined in 147/149 patients, we only included this cohort in the analysis of primary and secondary dengue. Accordingly, 49/147 (33.33%) had -primary dengue (DF = 40, DHF = 09) and 98/147 (66.67%) had secondary dengue (DF = 51, DHF = 47). There was no significant difference between the viral loads of those with primary and secondary dengue during early illness (*p* = 0.8609) (Fig. 2). There was no difference between the viral loads in primary and secondary dengue in those infected with different DENV serotypes. However, the number of patients infected with DENV1 (*n* = 32) and DENV3 (*n* = 23) who were included in the analysis was too small for a meaningful analysis. The incidence of DHF/DSS was higher in patients with secondary infections (47.9%) compared to those with primary infections (18.4%). Secondary infections were significantly associated with developing the DHF (*p* = 0.0006, Odds ratio 0.24; 95% CI 0.1028 to 0.5714).

### Association of viral loads with laboratory parameters

**Fig. 3:**
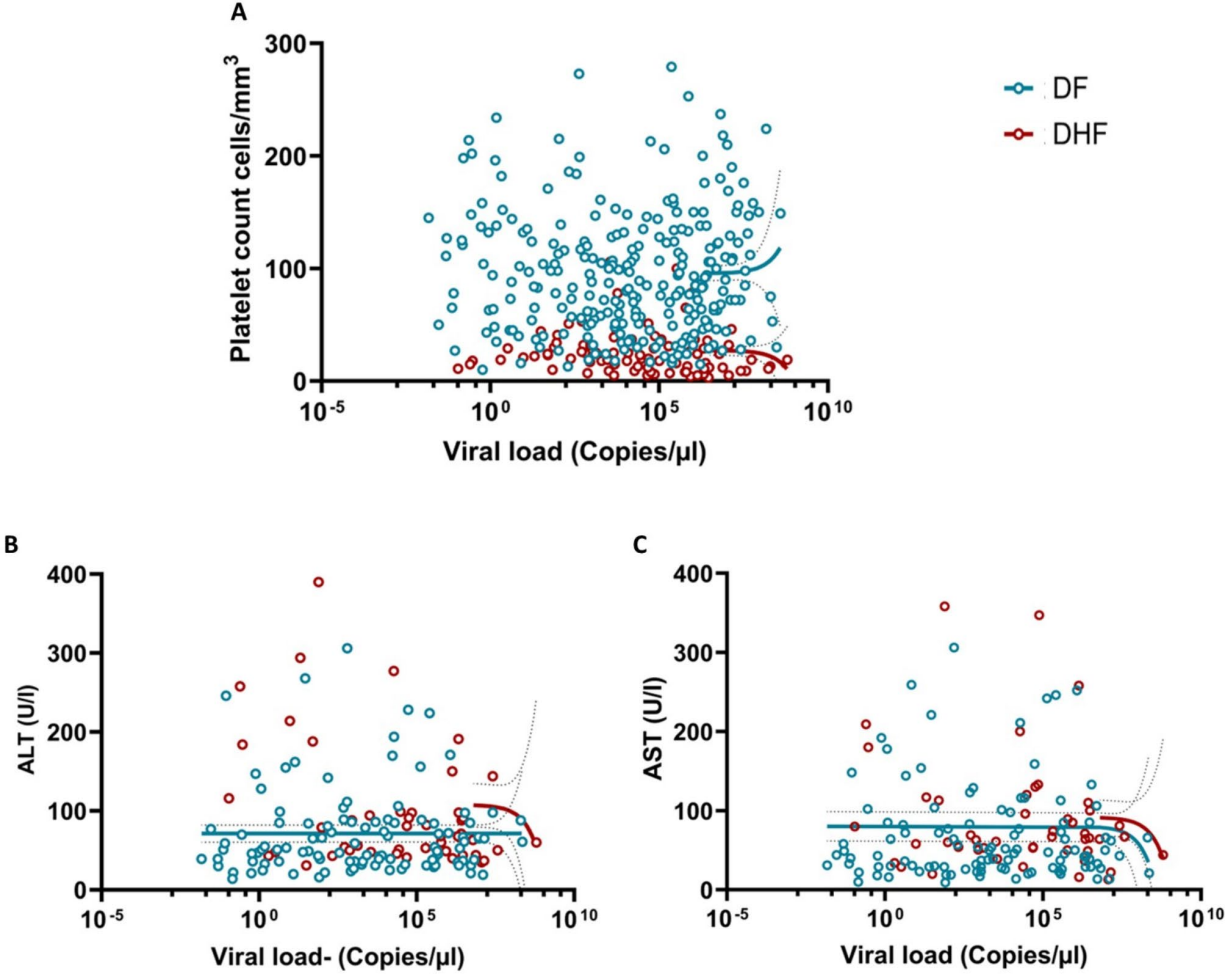
Association between the viral loads and the laboratory parameters in patients with varying severity of acute dengue. The correlation between the viral loads and the lowest platelet counts were assessed in patients with acute dengue with DF (*n* = 261) or DHF (*n* = 98). (**A**). Data of 3 patients who had co-infection are not included in the analysis. Viral loads were also correlated with ALT levels (**B**) and AST levels (**C**) in patients with DF (*n* = 107) and DHF (*n* = 52). Spearman rank order correlation coefficient was used to evaluate the correlation between the viral loads and AST and ALT. All tests were two sided

The viral loads in the febrile phase inversely correlated with the lowest platelet counts of those who progressed to develop DHF (Spearman’s *R*= -0.2393, *p* = 0.0.0182) (Fig. 3A), but not with their lowest leucocyte counts (Spearman’s *R*= -0.0012, *p* = 0.9908). No such association was seen in those with DF for platelet counts (Spearman’s *R* = 0.0118, *p* = 0.8508) (Fig. 3B) or leucocyte counts (Spearman’s *R*= -0.0410, *p* = 0.5111). Liver enzymes (AST and ALT) could only be done in 159 patients, as these investigations were not routinely done throughout the years in which the samples were collected. There was no correlation seen with the viral loads and ALT (Spearman’s *R*= -0.0125, *p* = 0.8766) (Fig. 3C) or AST (Spearman’s *R*= -0.0370, *p* = 0.6440) (Fig. 3D). However, a significant difference was observed in AST (*p* = 0.0066) and ALT (*p* = 0.0008) levels between those who developed DHF and DF.

### Viral loads and relationship with clinical disease severity at the time of recruitment based on WHO 2009 criteria

**Fig. 4:**
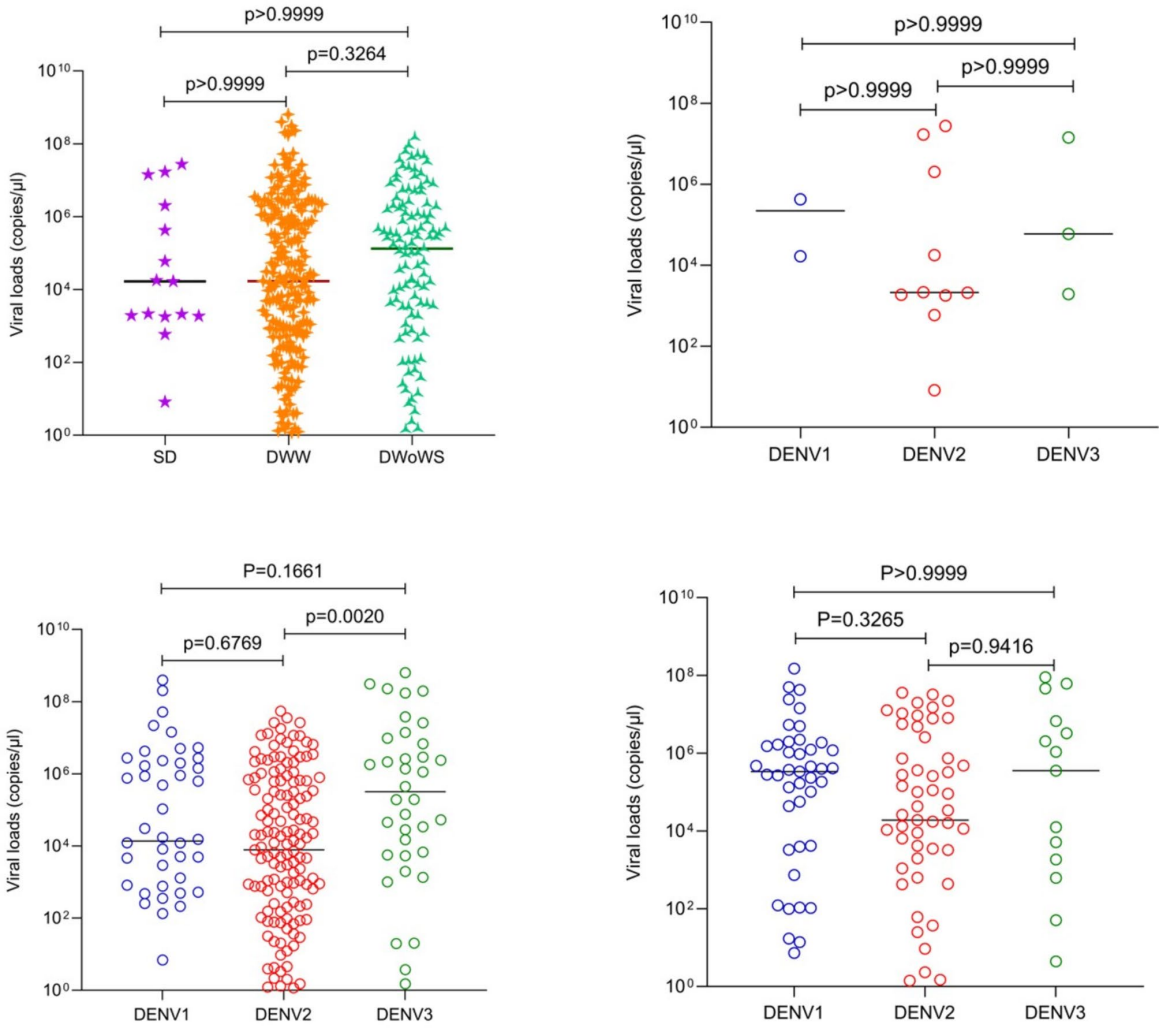
Viral loads in patients with varying clinical disease severity (classified according to the WHO 2009 disease classification) and infected with different DENV serotypes. Viral loads between patients with SD (*n* = 15), DWW (*n* = 231) and DWoWS (*n* = 113) were compared (**A**). Data of 3 patients who had co-infection are not shown. Viral loads of different serotypes in patients with SD (*n* = 15) (**B**), in those with DWW (*n* = 231) (**C**) and in those with DWoWS (*n* = 113) (**D**) were also compared. Kruskal-Wallis test was performed to compare the viral loads among the three groups of varying severity and the infected serotype. All tests were two sided. Data are presented as median values +/- interquartile ranges as appropriate and the p value is indicated

No significant difference was observed in the viral loads between those with SD, DWW and DWoWS (*p* = 0.2722) (Fig. 4A). However, significant differences were observed with the viral loads for different DENV serotypes in DWW (*p* = 0.0028) (Fig. 4B, C and D). Overall, the viral loads were lower with DENV2, compared to viral loads due to DENV1 and DENV3 in patients of all clinical disease categories.

## Discussion

In this study we have assessed the viral loads during early illness, over a period of five years in a large cohort of patients from Sri Lanka, who were followed up daily throughout the course of their illness, to determine their clinical disease severity. We used both the WHO 1997 and WHO 2009 to characterize disease severity and to determine if viral loads were predictive of disease severity, as different countries use different disease severity classification criteria. Using the WHO 1997 dengue disease classification, we found that there was a trend towards viral loads being higher in those who progressed to DHF when infected with DENV2 and DENV3, although such a trend was not seen when the WHO 2009 dengue disease classification was used. Therefore, the viral loads did not appear to associate with subsequent disease outcomes, irrespective of the disease classification used. However, previous studies using different clinical disease severity classifications have shown that viral loads at presentation do in fact associate with subsequent disease severity in children with low levels of DENV antibody titres [19]. A large study in Vietnam too showed that higher viral loads in the febrile phase was associated with an increased risk of hospitalization and plasma leakage, although the risk was modest [11]. Although we did not find viraemia levels associate with disease severity, the viral loads did inversely correlate with the extent of thrombocytopenia, as shown in previous studies [20].

We found significant differences in the viral loads in patients infected with different serotypes, with viral loads being highest with DENV3 and lowest with DENV2. As reported previously from a large study in Vietnam, and many other studies, we found that DENV2 was associated with lower viral loads, but with a higher risk of developing DHF [11, 21]. Higher viral loads for DENV1, followed by DENV3 has been shown in two previous studies from Vietnam [11, 12] further showing that the kinetics of viral loads significantly differ between the DENV serotypes during early illness. As certain genotypes of the DENV appears to associate with increased disease severity (cosmological strain of DENV2) and with certain neurological complications, it would be important to further evaluate the differences in viral loads and kinetics for these different DENV genotypes [22, 23]. Furthermore, these differences in the viral loads and NS1 antigen levels of different DENV serotypes, have significant implications when evaluating point-of care diagnostics and antiviral drugs for dengue. Therefore, in such evaluations, it would be important to carry out studies across different geographical regions and populations, which may have different circulating DENV serotypes and genotypes.

Although many studies do not show a statistically significant association with viral loads and clinical disease severity, many have shown a trend towards increased disease severity when infected with DENV2 [11], which had the lowest viral loads of the DENV serotypes. It is possibly due to greater immune evasive capacity by DENV2 infection, as strains of DENV2 have shown an enhanced ability to inhibit type 1 interferon signaling compared to other DENV serotypes [24]. Apart from these variations seen between DENV serotypes and particular genotypes, there is a huge individual variation between viral loads. Our data along with many other studies show several log differences of viral loads for different DENV serotypes in patients with varying disease severity during the febrile phase. Many factors such as the initial viral inoculum, the incubation period, previous immunity, and presence of comorbid illnesses have shown to affect viral loads [19, 25]. It was shown that the incubation period was shorter in those with secondary dengue compared to primary infection, supporting the role of antibody dependent enhancement in disease pathogenesis [25, 26].

Although we analyzed the differences in viral loads in the febrile phase in those with primary and secondary dengue, the sample size was small and only a few patients were infected with DENV1 and DENV3, to include in analysis. However, our results were similar to the large study done in Vietnam comparing viral loads in relation to immune status, which showed no difference in early illness [11]. A previous study done in Vietnam and in Singapore for evaluation of the antiviral effect of celgosivir showed that the viral loads were not in fact different in the early phase, but those with secondary dengue, cleared the plasma viraemia earlier than those with primary dengue [12, 27]. Therefore, although patients with secondary dengue have shorter incubation periods and possibly higher viral infection in FcγR bearing cells, due to antibody dependent enhancement, this is not reflected in the viraemia seen in serum. This possibly suggests that the viral loads and viral kinetics seen in serum may not necessarily reflect the viraemia within immune cells and tissues.

## Conclusions

In summary, although the degree of viraemia leads to severe disease, there is an important role played by the host-immune response to which could lead to resolution of infection or lead to immunopathogenesis. While use of antivirals would be an important treatment strategy for dengue, drugs that target certain immune mediators, such as leukotrienes, chymase, tryptase, platelet activating factor, growth factors, cytokines and other lipid mediators would be of potential benefit.

## Electronic supplementary material

Below is the link to the electronic supplementary material.

Supplementary Material 1

## Data Availability

All data is available in the manuscript, figures and the supporting files.

## References

[1] WHO. Ten threats to global health in 2019. In.: World Health Organization; 2019.

[2] Colon-Gonzalez FJ, Sewe MO, Tompkins AM, Sjodin H, Casallas A, Rocklov J, et al. Projecting the risk of mosquito-borne diseases in a warmer and more populated world: a multi-model, multi-scenario intercomparison modelling study. Lancet Planet Health. 2021;5(7):e404–14. 10.1016/S2542-5196(21)00132-7. 34245711 10.1016/S2542-5196(21)00132-7 PMC8280459

[3] WHO. Comprehensive guidelines for prevention and control of dengue fever and dengue haemorrhagic fever. SEARO Technical Publication Series. Volume 60. New Delhi, India: World Health Organization;: SEARO; 2011.

[4] Malavige GN, Jeewandara C, Ogg GS. Dengue and COVID-19: two sides of the same coin. J Biomed Sci. 2022;29(1):48. 10.1186/s12929-022-00833-y. 35786403 10.1186/s12929-022-00833-y PMC9251039

[5] Macias AE, Werneck GL, Castro R, Mascarenas C, Coudeville L, Morley D, et al. Mortality among hospitalized dengue patients with comorbidities in Mexico, Brazil, and Colombia. Am J Trop Med Hyg. 2021;105(1):102–9. 10.4269/ajtmh.20-1163. 33970884 10.4269/ajtmh.20-1163 PMC8274750

[6] Sangkaew S, Ming D, Boonyasiri A, Honeyford K, Kalayanarooj S, Yacoub S, et al. Risk predictors of progression to severe disease during the febrile phase of dengue: a systematic review and meta-analysis. Lancet Infect Dis. 2021;21(7):1014–26. 10.1016/S1473-3099(20)30601-0. 33640077 10.1016/S1473-3099(20)30601-0 PMC8240557

[7] Malavige GN, Jeewandara C, Ogg GS. Dysfunctional Innate Immune responses and severe dengue. Front Cell Infect Microbiol. 2020;10:590004. 10.3389/fcimb.2020.590004. 33194836 10.3389/fcimb.2020.590004 PMC7644808

[8] St John AL, Rathore APS. Adaptive immune responses to primary and secondary dengue virus infections. Nat Rev Immunol. 2019;19(4):218–30. 10.1038/s41577-019-0123-x. 30679808 10.1038/s41577-019-0123-x

[9] Bhatt P, Sabeena SP, Varma M, Arunkumar G. Current understanding of the pathogenesis of Dengue Virus infection. Curr Microbiol. 2021;78(1):17–32. 10.1007/s00284-020-02284-w. 33231723 10.1007/s00284-020-02284-w PMC7815537

[10] Troost B, Smit JM. Recent advances in antiviral drug development towards dengue virus. Curr Opin Virol. 2020;43:9–21. 10.1016/j.coviro.2020.07.009. 32795907 10.1016/j.coviro.2020.07.009

[11] Vuong NL, Quyen NTH, Tien NTH, Tuan NM, Kien DTH, Lam PK, et al. Higher plasma viremia in the Febrile phase is Associated with adverse dengue outcomes irrespective of infecting serotype or host Immune Status: an analysis of 5642 Vietnamese cases. Clin Infect Dis. 2021;72(12):e1074–83. 10.1093/cid/ciaa1840. 33340040 10.1093/cid/ciaa1840 PMC8204785

[12] Tricou V, Minh NN, Farrar J, Tran HT, Simmons CP. Kinetics of viremia and NS1 antigenemia are shaped by immune status and virus serotype in adults with dengue. PLoS Negl Trop Dis. 2011;5(9):e1309. 10.1371/journal.pntd.0001309. 21909448 10.1371/journal.pntd.0001309 PMC3167785

[13] Nainggolan L, Dewi BE, Hakiki A, Pranata AJ, Sudiro TM, Martina B, et al. Association of viral kinetics, infection history, NS1 protein with plasma leakage among Indonesian dengue infected patients. PLoS ONE. 2023;18(5):e0285087. 10.1371/journal.pone.0285087. 37130105 10.1371/journal.pone.0285087 PMC10153689

[14] Singla M, Kar M, Sethi T, Kabra SK, Lodha R, Chandele A, et al. Immune Response to Dengue Virus infection in Pediatric patients in New Delhi, India–Association of Viremia, Inflammatory mediators and monocytes with Disease Severity. PLoS Negl Trop Dis. 2016;10(3):e0004497. 10.1371/journal.pntd.0004497. 26982706 10.1371/journal.pntd.0004497 PMC4794248

[15] Perdomo-Celis F, Salgado DM, Narvaez CF. Magnitude of viremia, antigenemia and infection of circulating monocytes in children with mild and severe dengue. Acta Trop. 2017;167:1–8. 10.1016/j.actatropica.2016.12.011. 27986543 10.1016/j.actatropica.2016.12.011

[16] Silva T, Jeewandara C, Gomes L, Gangani C, Mahapatuna SD, Pathmanathan T, et al. Urinary leukotrienes and histamine in patients with varying severity of acute dengue. PLoS ONE. 2021;16(2):e0245926. 10.1371/journal.pone.0245926. 33544746 10.1371/journal.pone.0245926 PMC7864425

[17] WHO. Dengue guidelines, for diagnosis, treatment, prevention and control. In. Edited by (TDR) AjpotWHOWatSPfRaTiTD; 2009. 23762963

[18] Ministry of Health SL. Guidelines on management of dengue fever and dengue haemorrhagic fever in adults. National guidelines. Sri Lanka: Ministry of Health; 2012.

[19] Waggoner JJ, Katzelnick LC, Burger-Calderon R, Gallini J, Moore RH, Kuan G, et al. Antibody-dependent enhancement of severe disease is mediated by serum viral load in Pediatric Dengue Virus infections. J Infect Dis. 2020;221(11):1846–54. 10.1093/infdis/jiz618. 32236481 10.1093/infdis/jiz618 PMC7213574

[20] Pathak B, Chakravarty A, Krishnan A. High viral load positively correlates with thrombocytopenia and elevated haematocrit in dengue infected paediatric patients. J Infect Public Health. 2021;14(11):1701–7. 10.1016/j.jiph.2021.10.002. 34655984 10.1016/j.jiph.2021.10.002

[21] Vicente CR, Herbinger KH, Froschl G, Malta Romano C, de Souza Areias Cabidelle A, Cerutti Junior C. Serotype influences on dengue severity: a cross-sectional study on 485 confirmed dengue cases in Vitoria, Brazil. BMC Infect Dis. 2016;16:320. 10.1186/s12879-016-1668-y. 27393011 10.1186/s12879-016-1668-y PMC4938938

[22] Ngwe Tun MM, Muthugala R, Nabeshima T, Rajamanthri L, Jayawardana D, Attanayake S, et al. Unusual, neurological and severe dengue manifestations during the outbreak in Sri Lanka, 2017. J Clin Virol. 2020;125:104304. 10.1016/j.jcv.2020.104304. 32145478 10.1016/j.jcv.2020.104304

[23] Chan KWK, Watanabe S, Jin JY, Pompon J, Teng D, Alonso S, et al. A T164S mutation in the dengue virus NS1 protein is associated with greater disease severity in mice. Sci Transl Med. 2019;11(498). 10.1126/scitranslmed.aat7726. 10.1126/scitranslmed.aat7726 31243154

[24] Medina FA, Torres-Malave G, Chase AJ, Santiago GA, Medina JF, Santiago LM, et al. Differences in type I interferon signaling antagonism by dengue viruses in human and non-human primate cell lines. PLoS Negl Trop Dis. 2015;9(3):e0003468. 10.1371/journal.pntd.0003468. 25768016 10.1371/journal.pntd.0003468 PMC4359095

[25] Ben-Shachar R, Schmidler S, Koelle K. Drivers of inter-individual variation in Dengue viral load Dynamics. PLoS Comput Biol. 2016;12(11):e1005194. 10.1371/journal.pcbi.1005194. 27855153 10.1371/journal.pcbi.1005194 PMC5113863

[26] Riswari SF, Velies DS, Lukman N, Jaya UA, Djauhari H, Ma’roef CN, et al. Dengue incidence and length of viremia by RT-PCR in a prospective observational community contact cluster study from 2005–2009 in Indonesia. PLoS Negl Trop Dis. 2023;17(2):e0011104. 10.1371/journal.pntd.0011104. 36745606 10.1371/journal.pntd.0011104 PMC9901748

[27] Sung C, Wei Y, Watanabe S, Lee HS, Khoo YM, Fan L, et al. Extended evaluation of Virological, Immunological and Pharmacokinetic endpoints of CELADEN: a Randomized, Placebo-Controlled Trial of Celgosivir in Dengue Fever patients. PLoS Negl Trop Dis. 2016;10(8):e0004851. 10.1371/journal.pntd.0004851. 27509020 10.1371/journal.pntd.0004851 PMC4980036

